# Feasibility of Utilizing Spot-Scanning Proton Arc (SPArc) for Whole-Lung Irradiation: A Case Report

**DOI:** 10.1016/j.ijpt.2025.100750

**Published:** 2025-05-22

**Authors:** Peilin Liu, Lewei Zhao, Gang Liu, Xi Cao, An Qin, Di Yan, Xiaoqiang Li, Craig Stevens, Rohan Deraniyagala, Xuanfeng Ding

**Affiliations:** 1 Department of Radiation Oncology, Beaumont Health System, Royal Oak, MI 48084, USA; 2 Cancer Center, Corewell Health William Beaumont University Hospital, Tongji Medical College, Huazhong University of Science and Technology, Wuhan, 48073, PR China

**Keywords:** Proton arc, Whole-lung irradiation, Pediatric cancer

## Abstract

**Purpose:**

Photon radiotherapy is the conventional method in the treatment of bilateral whole-lung metastasis. However, uncertainties, longer delivery times, large lateral penumbra, and motion interplay limit intensity-modulated proton therapy (IMPT)’s use in bilateral lung metastases. To overcome such limitations in IMPT, this study explores the feasibility of using a novel proton therapy technique, Spot-scanning Proton Arc (SPArc) therapy, to improve the dose sparing to the heart and other healthy tissue for this pediatric patient compared to the volumetric modulated arc therapy (VMAT) and IMPT.

**Patients and Methods:**

A 13-year-old patient with a malignant neoplasm of bone and articular cartilage, presenting with bilateral whole-lung metastasis, received whole-lung irradiation of 15 Gy in 10 fractions using VMAT. For comparative analysis, plans were generated using IMPT and SPArc.

**Results:**

The study showed that SPArc was superior in sparing the heart and enhancing delivery efficiency compared to both VMAT and IMPT. The mean heart dose was 5.41 Gy for SPArc, 8.48 Gy for IMPT, and 9.56 Gy for VMAT. D50 of the heart was 3.06 Gy for SPArc, 9.13 Gy for IMPT, and 9.12 Gy for VMAT. The integral body dose was 137 Gy·L in VMAT,189 Gy·L in IMPT, and 98 Gy·L in SPArc.

**Conclusion:**

Spot-scanning proton arc demonstrated effective heart sparing and lower body-integral dose for whole-lung irradiation. Delivery simulations suggested improved efficiency compared with IMPT.

## Introduction

Whole lung irradiation (WLI) is pivotal in treating pulmonary metastasis in pediatric patients.^1^, ^2^ The 5-year overall survival with and without WLI was 61% and 49%, respectively.^3^, ^4^ WLI significantly improves local control of lung disease and leads to a trend toward better progression-free survival.^5^ The overall survival rate and relapse-free survival are 78% and 72% in children with favorable histology tumors in the National Wilms Tumor study-3.^6^ However, radiation therapy, particularly to the heart, can lead to a range of complications impacting the pericardium, myocardium, endocardium, cardiac valves, conduction system, and coronary arteries.^4^, ^7^, ^8^ These cardiovascular disorders are a leading cause of premature mortality among long-term cancer survivors.^1^ The 20-year congestive heart failure rate in the National Wilms Tumor was 4.4% after initial treatment and 17.4% after the first or subsequent relapse.^4^ Further, a study of 4122 French-British cancer survivors, monitored for a median of 26 years, revealed that the adjusted relative risk of cardiac mortality was significantly elevated (RR 7.4) after receiving heart radiation doses exceeding 5 Gy. This study was among the first to establish a link between low to intermediate cardiac doses (5-15 Gy) and increased cardiovascular mortality in childhood cancer survivors.^8^ Thus, potential cardiac toxicity from WLI must be acknowledged, and improved radiation techniques are highly desirable.^9^

Proton therapy’s physical properties enable significant dose reduction to surrounding normal tissues, a benefit particularly relevant for children.^10^ Recently, spot-scanning proton arc (SPArc) therapy was introduced into radiation oncology as a concept in 2016, allowing gantry rotation while delivering proton spots and switching energies.^11^, ^12^ The previous investigation reported significant improvement in the dosimetric outcome in various clinical indications such as lung,^13^ breast,^14^ prostate,^15^ head and neck cancers.^16^ This is the first study to investigate the feasibility of utilizing such a novel treatment technique in WLI for a pediatric patient with bilateral pulmonary metastasis through the dosimetric comparison, treatment delivery, and interplay effect simulation.

## Materials and methods

### Case description of clinical details and treatment modalities

**Figure 1:**
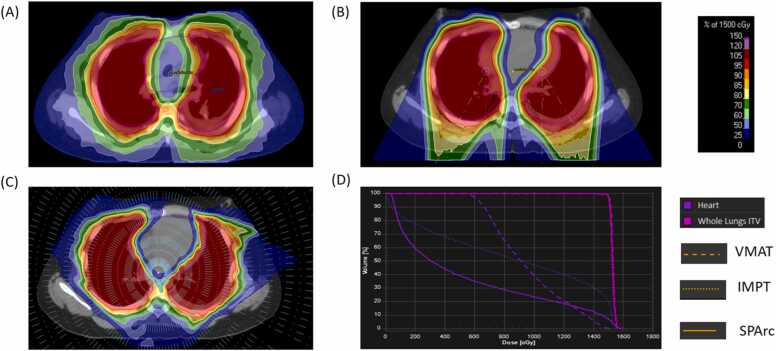
The SPArc versus IMPT and VMAT treatment plan. (A) SPArc plan dose, (B) IMPT plan dose, (C) VMAT plan dose, and (D) DVH comparison. Abbreviations: DVH, dose-volume histogram; IMPT, intensity-modulated proton therapy; SPArc, spot-scanning proton arc therapy; and VMAT, volumetric modulated arc therapy.

This study, approved by the Institutional Review Board, analyzed patient data from the Department of Radiation Oncology at William Beaumont Hospital. The patient, a 13-year-old teenager diagnosed in April 2021 with malignant bone and articular cartilage neoplasm and bilateral whole-lung metastasis (Figure 1A), had their personal information anonymized. In June 2021, the patient underwent 15 Gy of WLI in 10 fractions with volumetric modulated arc therapy (VMAT).

The lung internal target volume was defined as 1919.75 cm³, encompassing the maximum lung expansion volume on 4D computed tomography simulation scans.^1^ Given the substantial size of the target, VMAT delivered a considerable dose to both the heart and the overall body. To evaluate alternatives, IMPT and SPArc plans were generated.

### Treatment planning and dosimetric plan quality comparison

For the clinical VMAT plan, 3 full arcs utilizing a 6 MV beam from the Elekta Versa HDTM were employed. The IMPT plan (Figure 1B) was generated using a dual-isocenter and 4 treatment fields with the single field optimization in the RayStation (Stockholm, Sweden). The SPArc plan was generated through a published in-house developed optimization algorithm and implemented in RayStation through scripting.^12^ SPArc employed a full 360-degree arc trajectory at 2.5-degree intervals from a single isocenter (Figure 1C).

Both IMPT and SPArc plans incorporated the same robustness settings (±5 mm setup, ±3.5% range) and used 3 mm dose grids. Each plan delivered 15 Gy in 10 fractions, requiring at least 98% of the internal target volume to receive the full prescription. Comparative evaluations, including heart dose-volume histograms and integral body dose assessments in VMAT, IMPT, and SPArc nominal plans.

### Delivery time calculation

The delivery efficiency for both IMPT and SPArc was simulated using a previously published machine delivery sequence model for the IBA ProteusONE machine.^17^ The VMAT delivery time was obtained from the LINAC machine’s log file.

### Interplay effect evaluation

A 4D computed tomography data set comprising 10 phase images was utilized to simulate a 4-second patient breathing cycle. The interplay effect was evaluated using a 4D dynamic dose accumulation approach.^18^ For each treatment fraction, the dynamic dose calculation involved accumulating the dose from each phase image onto the reference phase (50%) using deformable image registration. The D98 metric was used to assess target coverage.

## Results

### Result of diagnostic assessment

As illustrated in Figure 1, the patient's SPArc plan was evaluated alongside the IMPT and VMAT plans, with their respective optimized parameters detailed in Table 1. Notably, SPArc's total monitor units were lower than IMPT's, although SPArc utilized a slightly higher number of energy layers and spots.

**Table 1:** Optimized plan parameters comparison.

Plan	Number of energy layers	Number of spots	Total MUs
VMAT	Not applicable	Not applicable	721
IMPT	119	24 743	4050
SPArc	134	27 407	3822

**Abbreviations:** MU, monitor unit; VMAT, volumetric modulated arc therapy; IMPT, intensity-modulated proton therapy; SPArc, spot-scanning proton arc.

### Result of dose metrics comparison

SPArc demonstrated a notable reduction in dose to healthy tissue compared to IMPT and VMAT, while maintaining comparable coverage of clinical target volumes (Table 2). Specifically, the mean heart dose in the SPArc plan was 5.41 Gy, significantly lower than 8.48 Gy in IMPT and 9.56 Gy in VMAT. In terms of D50 (the dose received by at least 50% of the heart's volume), SPArc achieved 3.06 Gy, substantially less than IMPT's 9.13 Gy and VMAT's 9.12 cGy. Furthermore, the integral body dose was remarkedly reduced in SPArc (98 Gy·L) compared to VMAT (136 Gy·L) and IMPT (112 Gy·L).

**Table 2:** Dose metrics statistics and delivery time comparison among VMAT, IMPT, and SPArc.

Comparison index		VMAT	IMPT	SPArc
ITV	Max dose (cGy)	1594	1623	1599
	Mean dose (cGy)	1600	1526	1530
	D98 (cGy)	1494	1500	1500
Heart	Max dose (cGy)	1541	1596	1595
	Mean dose (cGy)	956	848	541
	D50 (cGy)	912	913	306
Body-integral dose (Gy·L)		136	112	98
Irradiation times^a^ (s)		N/A	746	973
Total delivery time^b^ (s)		317	1046	985

**Abbreviations:** VMAT, volumetric modulated arc therapy; IMPT, intensity-modulated proton therapy; SPArc, spot-scanning proton arc; ITV, internal target volume.

a The irradiation time for proton beam therapy includes the delivery of all the spot and energy layer at a fixed-beam angle. It includes spot drill time, spot-scanning time, energy layer switching time, and burst to switch time.

b Total delivery time includes the irradiation time, the estimated iso shift workflow, imaging verification time,^14^ as well as the gantry rotation time.

### Result of delivery efficiency comparison

The LINAC log file indicated that VMAT completed its 3-arc delivery in 317 seconds. In comparison, the total IMPT treatment delivery for WLI is approximately 1046 seconds. Conversely, SPArc achieves dynamic arc delivery in a total time of 985 seconds.^17^ This represents a 5.83% reduction in treatment delivery time compared to the 4-field IMPT approach.

### Result of interplay effect evaluation

**Figure 2:**
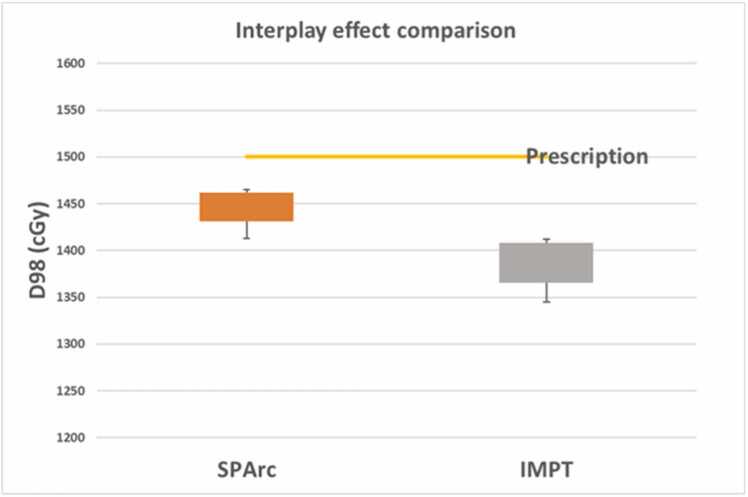
Comparison of interplay effect between SPArc and IMPT. Abbreviations: IMPT, intensity-modulated proton therapy; SPArc, spot-scanning proton arc therapy.

SPArc achieved a mean D98 dose of 14.45 ± 0.19 Gy, surpassing the 13.85 ± 0.26 Gy achieved by IMPT (Figure 2). Statistically, SPArc demonstrated superior target coverage compared to IMPT, as indicated by a significant difference in a *t* test (*P* value < .01).

## Discussion

A fundamental principle of radiation therapy is to minimize the dose to nontarget normal tissues, which is critical to pediatric patients. The tumor rarely invades the heart in children with lung metastasis. However, it is unavoidable during WLI because of its anatomic proximity to the lungs.^4^, ^19^, ^20^, ^21^ Such complicated geometry makes conventional radiation therapy less effective in cardiac structure sparing. Thus, we reported a first feasibility study of utilizing a novel treatment technique, SPArc, for such a challenging case who received WLI in our institution. The result demonstrated a significant heart dose and body-integral dose sparing compared to VMAT, multifield IMPT, and SPArc. The study found that SPArc also reduces the patient’s total body-integral dose. Such dosimetric improvement has the potential to reduce normal tissue complications such as cardiac mobility^22^ and secondary malignancy,^23^ which is critical to childhood cancer survivors. Additionally, the study found that the demonstrated treatment efficiency of SPArc could be comparable to that of IMPT. Furthermore, this case study indicated that SPArc was able to mitigate the intrafractionation motion uncertainties, which agrees with the previous finding in the mobile target treatment such as advanced staged and early-stage non–small cell lung cancer treatment.^24^ These findings indicated that free-breathing conditions with a 4D scan could provide sufficient plan robustness in terms of the target coverage in the SPArc WLI. However, this is a case study. More patient data are needed to provide more concrete data and evidence to support the recommendation.

## Conclusion

SPArc technique in whole-lung irradiation offers substantial dosimetric benefits over VMAT and IMPT, particularly in reducing cardiac and overall body exposure. Additionally, SPArc simplifies the clinical workflow and effectively manages the interplay effect compared to IMPT.

## Ethics

The study is approved by IRB 2017-455, and consent is waived by the participant.

## Author Contributions

Conceptualization: X.D., R.D.; Data curation: P.L.,L.Z.,G.L., X.D.; Formal analysis: P.L., L.Z.; Investigation: P.L., L.Z.,G.L.,X.C., A.Q.,D.Y.,C.S., R.D., X.L., X.D.; Methodology: X.D., P.L.,L.Z.,G.L.; Supervision: X.D., R.D., C.S.; Writing: P.L., L.Z.,G.L.,X.C.,X.D.

## Declaration of Conflicts of Interest

The authors declare the following financial interests/personal relationships, which may be considered as potential competing interests: Xuanfeng Ding reports that financial support was provided by Ion Beam Applications SA. Xuanfeng Ding reports that financial support was provided by Corewell Health William Beaumont University Hospital. Xuanfeng Ding reports a relationship with Ion Beam Applications SA that includes speaking and lecture fees. Xuanfeng Ding has a patent licensed to Ion Beam Application SA. If there are other authors, they declare that they have no known competing financial interests or personal relationships that could have appeared to influence the work reported in this paper.
