# Supplementary material for: Feasibility of Utilizing Spot-Scanning Proton Arc (SPArc) for Whole-Lung Irradiation: A Case Report
Source: Int J Part Ther. 2025 May 22;16:100750. doi: 10.1016/j.ijpt.2025.100750 (PMC12162079; doi:10.1016/j.ijpt.2025.100750)
Supplement: Supplementary file 1 — Supplementary material [file mmc1.pdf]

Supplemental document

1. Contour criterion: Contouring was performed on the average scan for the target with <1.0 cm diaphragmatic motion[1].

2. Plan detail:

1） VMAT beam alignment:

Beam number	Gantry range (°)	Rotation direction	Collimator angle (°)	Couch angle (°)
1	181-179	Clockwise	10	0
2	178-182	Counterclockwise	350	0
3	183-177	Clockwise	5	0

2) IMPT beam alignment

Beam number	Gantry angle (°)	Couch angle (°)	Range shifter
1	180	180	4cm
2	150	180	4cm
3	148	0	4cm
4	180	0	4cm

3） Three treatment plan dose calculation algorithm and dose grid

Treatment modality	Dose calculation algorithm	Dose grid
VMAT	CCC	$0.3cm^3*0.3cm^3*0.3cm^3$
IMPT	Monte Carlos	$0.3cm^3*0.3cm^3*0.3cm^3$
SPArc	Monte Carlos	$0.3cm^3*0.3cm^3*0.3cm^3$

4) Similar optimization objective functions of OARs have been used in three modalities different treatment system and technique with the same clinical dose constrain to OARs and target coverage requirement.

3. Robustness evaluation of proton plans : Each proton plan satisfied the robustness requirement that, in the worst-case scenario, at least 95% of the prescription dose is delivered to 95% of the target volume[5,6]. The V95% in the worst-case scenario for SPArc was 99.68%, demonstrating superior robustness compared to IMPT, which was 97.49% (Figure S1).

4. Section of Interplay effect and evaluation:

   Method in detail:

   The interplay effect was simulated by assigning proton spots assigned into different respiratory phases based on the delivery sequence. More specifically, 4D CT contains 10 phase images. Assuming the patient normal breathing cycle is 4s. We could put the spots into different phases based on irradiation sequence of the proton system which is based on a validated IBA ProtuesONE model. Then, we calculate the dose on each phase and accumulate dose from each phase into the T50 CT to simulate the interplay effect dose distribution. Such interplay effect evaluation methods have been published by numerous groups and considered as an estimation tool for the research and clinical application.

   Discussion:

   Unique physical property of proton beam- Bragg peak, introduces significant challenges in managing respiratory motion during whole lung irradiation (WLI). The interplay effect could lead to significant dose perturbations in intensity-modulated proton therapy (IMPT), risking underdosing of moving targets or overdosing of critical structures like the heart. While strategies such as 4DCT-based internal target volume (ITV) delineation or 4D robust optimization mitigate these risks, they often result in expanded irradiated volumes or compromised nominal plan quality[2–4]. Benefiting by the number of control points, SPArc may have the ability to reduce interplay while maintaining cardiac sparing. This innovation holds particular promise in pediatric WLI, where motion robustness and long-term toxicity reduction are paramount.

5. The result of MFO plan dosimetric parameters:

ITV	Max dose (cGy)	1675
	Mean dose (cGy)	1577
	D98 (cGy)	1500
Heart	Max dose (cGy)	1612
	Mean dose (cGy)	703
	D50 (cGy)	615
Body integral dose (Gy·L)		95.61

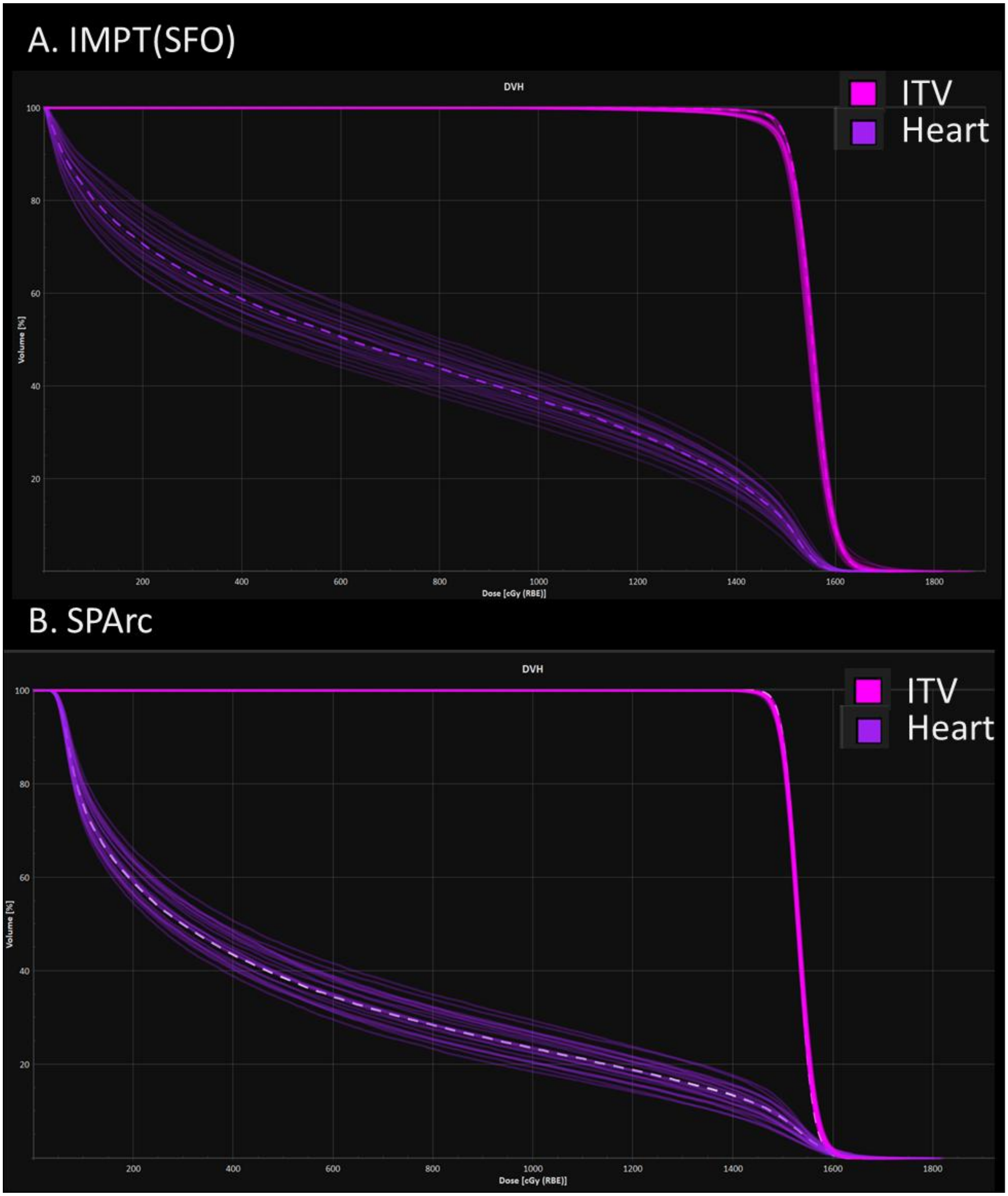
A. IMPT(SFO)
DVH
ITV
Heart
Volume [%]
100
80
60
40
20
200
400
600
800
1000
1200
1400
1600
1800
Dose [cGy (RBE)]
B. SPArc
DVH
ITV
Heart
Volume [%]
100
80
60
40
20
200
400
600
800
1000
1200
1400
1600
1800
Dose [cGy (RBE)]

Figure S1. The comparison of robustness evaluation between IMPT and SPArc

Reference:

1. Cunningham DA, Breen WG, Johnson JE, et al. Proton Whole-Lung Irradiation: Initial Report of Outcomes. *International Journal of Radiation Oncology*Biology*Physics*. 2023;115(4):866-872. doi:10.1016/j.ijrobp.2022.10.001

2. Cao X, Liu P, Gao X shu, et al. Redefine the Role of Proton Beam Therapy for the Locally-Advanced Non-Small Cell Lung Cancer Assisting the Reduction of Acute Hematologic Toxicity. *Front Oncol*. 2022;12:812031. doi:10.3389/fonc.2022.812031

3. Liu Y, Liu P, Gao XS, et al. Dosimetric comparison of IMPT vs VMAT for multiple lung lesions: an NTCP model-based decision-making strategy. *Medical Dosimetry*. Published online July 2024:S0958394724000293. doi:10.1016/j.meddos.2024.06.001

4. Liu C, Sio TT, Deng W, et al. Small-spot intensity-modulated proton therapy and volumetric-modulated arc therapies for patients with locally advanced non-small-cell lung cancer: A dosimetric comparative study. *J Applied Clin Med Phys*. 2018;19(6):140-148. doi:10.1002/acm2.12459

5. Newpower MA, Chiang B, Ahmad S, Chen Y. Spot delivery error predictions for intensity modulated proton therapy using robustness analysis with machine learning. *J Applied Clin Med Phys*. 2023;24(5):e13911. doi:10.1002/acm2.13911

6. Liu P, Gao X shu, Wang Z, et al. Investigate the Dosimetric and Potential Clinical Benefits Utilizing Stereotactic Body Radiation Therapy With Simultaneous Integrated Boost Technique for Locally Advanced Pancreatic Cancer: A Comparison Between Photon and Proton Beam Therapy. *Front Oncol*. 2021;11:747532. doi:10.3389/fonc.2021.747532
